# Caregiver experiences with accessing sickle cell care and the use of telemedicine

**DOI:** 10.1186/s12913-022-07627-w

**Published:** 2022-02-22

**Authors:** Seethal A. Jacob, Roua Daas, Anna Feliciano, Julia E. LaMotte, Aaron E. Carroll

**Affiliations:** 1 Center for Pediatric and Adolescent Comparative Effectiveness Research, Indiana University, HITS Building, 410 W. 10th St, Suite 2000A, Indianapolis, IN 46202 USA; 2 Division of Pediatric Hematology Oncology, Department of Pediatrics, Riley Hospital for Children, Indianapolis, IN 46202 USA; 3 Indiana University School of Medicine, Indianapolis, IN 46202 USA

**Keywords:** Telemedicine, Telehealth, Sickle cell disease, Pediatric, Access to health care

## Abstract

**Background:**

Sickle cell disease (SCD) is associated with a wide range of complications. However, a multitude of barriers prevent SCD patients from receiving adequate healthcare, including difficulties with transportation and lack of provider knowledge about disease sequelae. Importantly, studies have demonstrated the benefits of telemedicine in addressing barriers to healthcare. While previous studies have identified barriers to care through quantitative methods, few studies have explored barriers which affect the pediatric SCD patient population in the Midwest, wherein the geographical landscape can prohibit healthcare access. Furthermore, few studies have established acceptability of telemedicine among caregivers and patients with SCD.

**Methods:**

This study aims to increase understanding of barriers to care and perceptions of telemedicine by caregivers of pediatric SCD patients in a medically under-resourced area in the Midwest. Researchers conducted semi-structured interviews with caregivers of children with SCD. The interviews were audio-recorded and transcribed. Thematic analyses were performed.

**Results:**

Researchers interviewed 16 caregivers of 15 children with SCD. Thematic analyses of the interview transcripts revealed four broad themes regarding caregiver burden/stress, both facilitators and barriers to SCD healthcare, and general thoughts on the acceptability/usefulness of telemedicine.

**Conclusion:**

This qualitative study describes common burdens faced by caregivers of SCD, barriers to and facilitators of SCD care in the Midwest, and caregiver perceptions of the usefulness and efficacy of telemedicine for SCD care.

## Introduction

Sickle cell disease (SCD) is the most common inherited hematologic disease in the world, disproportionately affecting African Americans in the United States [1]. One out of every 365 African American newborns is diagnosed with SCD, many of whom are born into low-income families [1, 2]. The complications of SCD are numerous, and include severe pain, acute chest syndrome, and stroke [3]. However, improvements in preventative therapies have increased the life span for patients with SCD, with more than 93% of children with SCD surviving to adulthood [4]. Improved patient outcomes though, are contingent on SCD patients receiving timely evidence-based medical care, including regular patient evaluations with hematologists, disease-specific education, psychosocial care, and genetic counseling [5–7].

Despite the complexity of care required by SCD patients, many barriers prevent equitable access to healthcare [1, 8, 9]. Children with SCD experience longer than average emergency department (ED) wait times, difficulty with transportation, and insufficient availability of primary care providers [10]. Additionally, primary care providers report having inadequate SCD expertise, feeling uncomfortable with pain management, and other SCD-related care for prevention and treatment [11, 12]. Comprehensive Sickle Cell Centers are more commonly located in major metropolitan areas, creating additional challenges for families living in rural or under-resourced areas [13].

While previous studies have identified barriers to accessing care for adults with SCD, less is understood about the barriers that affect the pediatric SCD patient population [14]. Furthermore, there is limited data regarding barriers for those living in the Midwest, specifically in rural and medically under-resourced communities [1, 15, 16]. Telemedicine, the remote provision of medical care using real-time audio-visual consultation, is a valuable solution to addressing physician shortages and geographic barriers faced by patients and has proven successful for consultations in a variety of pediatric specialties [17]. However, information on the value of telemedicine exclusively for SCD patients is minimal in the existing literature, particularly for children with SCD [18–20]. Additionally, attitudes of caregivers and patients with SCD regarding the use of technology, such as telemedicine, for addressing these barriers are not well established [21, 22].

Semi-structured interviews have proven to be a powerful tool for providers to gain a deeper understanding of patient experiences [23]. This study aims to increase the understanding of both facilitators and barriers to care, as well as perceptions of telemedicine by caregivers of pediatric SCD patients in a medically under-resourced area in the Midwest.

## Methods

### Participants/procedure

Caregivers of pediatric patients with SCD seen at Riley Hospital for Children in Indianapolis, Indiana and live greater than 60 minutes from the hospital were contacted by the research team via phone or email. Those who did not meet these criteria were excluded. A total of 31 caregivers were asked to participate in a 30-min interview to understand facilitators and barriers to accessing sickle cell care, as well as acceptability of telemedicine for SCD care. Sixteen caregivers agreed to participate (response = 52%). The interviews were conducted by 2 members of the research team via Zoom or telephone, depending on the participant’s preference. At the beginning of the interview, the researcher and participant went over the study information sheet together, after which a standard consent procedure was conducted. Participants were compensated with a gift card for their time. This study was approved by the Indiana University Institutional Review Board.

### Measure/data collection/analysis

The semi-structured interviews were conducted between March and October of 2021 and were audio-recorded. Interview questions were developed from literature review, including adaption of questions related to expectations and pragmatic barriers from the Barriers to Care Questionnaire (BCQ) by Seid et al. [24]. The interviewer took notes and completed a field note following each interview.

Each interview was transcribed and subsequently coded by two authors. We used a thematic approach to analysis [25]. A codebook was created based on findings from literature review and refined/expanded with re-occurring themes encountered during review of transcripts. Example codes included barriers to care, facilitators of care, and acceptability of telemedicine. Any discrepancies were reviewed by the remaining authors. The authors were all in agreement that the completed interviews reached theoretical saturation [26], providing adequate representation of attitudes given the emergence of common themes with little new variation.

## Results

### Participants

A total of 16 caregivers of 15 children with SCD were interviewed (Table 1). Fourteen of the caregivers identified as female and as the primary caregiver. The majority of caregivers identified as Black (*n* = 11). On average, caregivers reported traveling 115 minutes one-way to receive SCD care. Fourteen of sixteen caregivers had participated in some model of telemedicine previously (e.g., hub-and-spoke or direct-to-consumer). Interviews lasted approximately 25 minutes (range 12–58 min).

**Table 1:** Participant Demographics

	*N* (%)
Primary caregiver (16 caregivers)	
Identifies as the primary caregiver of a child with SCD	16 (100%)
Identifies as the primary caregiver of more than one person	12 (75%)
Sex of Caregiver	
Male	2 (13.3%)
Female	13 (86.7%)
Race of Caregiver	
Black	11 (68.8%)
White	5 (31.3%)
Race of Child	
Black	15 (100%)
White	0 (0%)
Genotype of child with SCD	
Hb SS	10 (66.7%)
Hb SC	4 (26.7%)
Hb SB0	1 (6.7%)
Distance from Riley Hospital (mode)	
Time	60, 75, 90 mins
Distance	70, 146, 151 miles

*Abbreviations:* SCD Sickle Cell Disease

Thematic analyses of the interview transcripts revealed 4 broad themes regarding caregiver burden/stress, barriers or facilitators to healthcare accessibility for children with SCD and their families, as well as usefulness of telemedicine (Table 2).

**Table 2:** Themes and Associated Caregiver Quotes

Codes/Themes			Quotes
**Caregiver Effects**	**Grief/acceptance of diagnosis** (*n* = 9)		“It first started out, it was very, very difficult to grasp like knowing that my kid would have to be on medicine for the rest of her life” “I was young, so it was very difficult for me because I did have my son a year before her when she did not have sickle cell. So, I was a little bit confused as to how because, like I said, I was young at the time, so I didn’t know as much as I know now.”
	**Burden/stress** (*n* = 12)		“So it was just a lot to grasp and learn within the first year I would say... as far as making sure that like I said doing everything that I’m supposed to be doing and it was just a lot to take on with a newborn and having two other kids” “As I’ve gotten older, it has gotten manageable, but it is extremely overwhelming sometimes, taken the circumstances. It gets overwhelming at times.” “I have [chronic medical condition]. So, when I’m sick and she’s sick at the same time, it is a lot…I don’t have anybody else to really take of her. So, sometimes I have to cut my visits short…to get the care she needs, as well.”
	**Caregiver’s mental health** (*n* = 6)		“I would say I was young when I had [the patient], and it was really difficult for me at times. So, my mom would tell me you have to build yourself up. You have to be able to not cry and let her see you cry. You have to be strong. And so now, as I’m older, I have learned to suppress my emotions around her..., but on the inside you could really be breaking down.”
	**Guilt/responsibility** (*n* = 5)		“I was really sad because I know that he’s going to have to deal with it for the rest of his life, and there’s nothing that I can really do, and then I felt kind of responsible for it because I didn’t know that I had the trait, and I didn’t know that his father had the trait, so I kind of felt responsible as to like it was my fault that my son has this disease.”
**Barriers to Access**	**Caregiver Level**	**Financial/Missing work** (*n* = 11)	“It’s hard. I am a single parent…on my own, so I’ve lost jobs because of it because sometimes he’s sick, and me having to call off so much. So, I mean, it’s financially hard, but I mean, I wouldn’t, I mean, if I had to do it all over again I wouldn’t change anything. I love my son.” “…[My child] just spent…weeks in the hospital, and I was really worried because I just started a new job... there have been times in the past where I’ve lost jobs or gotten written up because I was at the hospital.”
		**Transportation** (*n* = 6)	“When we first found out [about diagnosis] I didn’t have [transportation] so I had to depend on people or even call like Medicaid to get a ride back and forth …[a]nd then it also made it difficult back.”
		**Missing school** (*n* = 11)	“It can be [difficult] because he has to miss school on top of the days that he is sick, so he misses a lot of school.”
	**System Level**	**Location** (*n* = 12)	“The fact that you’re about an hour and a half away would be the biggest obstacle for us but we just do it.” “The distance gets a little much, like in trying to, our family doesn’t live around here, and so with our daughter, it’s always trying to find a place for her to go.”
		**Accessibility** (*n* = 7)	“It’s always hard when I take him to the local emergency room, and then they decide they’re not really equipped to deal with him because there’s no hematologist, and then he has to be transferred to the hospital that’s 45 or 50 min away. And then, of course, I have to drive there just so when we’re released, we can have a way home. [Even though] I would want to be in the ambulance with my son just because I’m always worried, and I don’t want him to feel like he’s alone and I’m not there.”
		**Frequency of visits** (*n* = 4)	“I believe when [my child] first came home or the first couple of years he was home, I think he had about six appointments or seven appointments in 1 month each month. So, it was really, really tough to even have my own life and try to work or do anything or something else with the other kids.”
	**Healthcare Providers Level**	**Provider Knowledge/Bias** (*n* = 9)	“The first time I ever took my son to the hospital the ER doctor told me that my son couldn’t have sickle cell because I was white.” “So if they’re to the point where they’re going to the ER and they are going to have to have meds, [and] more doctors realize [d] that, and more people were educated on sickle cell …, they would understand that they’re not there seeking drugs.”
**Barrier or Facilitator**	**Provider to patient communication** (*n* = 10)		“And I just felt like at that time they just looked at me as a young mother and didn’t look at me as [the patient]‘s advocate. And then they didn’t want to listen to me when I told them that I knew my son” “They communicated with me. They sent me little packets on sickle cell disease, what to expect. It was a lot of stuff that I did receive from the Sickle Cell Association when I did find out she did have sickle cell.”
**Facilitators to Access**	**Healthcare system support**	**Convenience** (*n* = 14)	“Because hematology is not the only specialist we see … They try to make it a lot easier to not have to do the back and forth. So they try to combine appointments within the same day as other specialist… They make it a lot easier for me …” “[Travel for appointments] has actually been manageable for us, only because with us being in another city, they’ll book all the appointments together. Sometimes we might be there for … a doppler, and then see the [Ophthalmologist] for her eyes, and then we get the blood drawn, and all that.”
		**Hospital addressing barriers** (*n* = 6)	“[The hospital] did pay 1 month’s worth of my rent for me to make sure that everything was going to be okay” “The hospital itself has provided so much mental support, it is astounding. They will comment, like your nurses or your doctors will say...go get some air...I’m guessing they want to see the parents’ okay, even though the child may not be doing so good or going through things. They will come in and try and make sure that the parent is still eating, the parent is getting sleep... So, I would say the hospital itself mentally is a big help for me.”
		**Access to staff** (*n* = 9)	“Like I said [nurse coordinator]... has been a big help... I don’t have to call the front desk to be able to get ahold of them. I can literally just call the on-call phone that [nurse coordinator] carries. And if I have any type of questions, she can either give me the answer or she will ask [the doctor] and then get back with me. Typically, it’s literally within no more than 20, 30 min that she gets back with me. So it’s very, very smooth.”
		**Insurance Support** (*n* = 6)	“I try to utilize, from her insurance...someone comes from the medical place and picks us up and takes us down there.”
	**Community support**	**Family/friend Support** (*n* = 10)	“My mom has pretty much taken over the bulk of the load with [my child’s] education, so the e-learning...my mom is pretty much watching her throughout the day to make sure she’s following along”
**Telemedicine**	**Pros**	**Distance** (*n* = 8)	“We don’t have to travel out because that is a three-hour drive there and 3 h back. That’s 6 h...So, that was the most challenging is having to keep traveling there just to talk to a doctor when you can actually do it how we’re doing it now. So, the telemedicine actually just made it easier.”
		**Flexibility for Visits** (*n* = 10)	“I don’t want to do telehealth for a sick visit. But telehealth for other visits I’m okay with that.” “Now, if an outbreak happened or something, then, yeah, we could do it.”
	**Cons**	**Access to technology** (*n* = 4)	“A lot of times we do it on our phone and if you receive a call or something, you’re kicked out. Then they have to get back on and redo everything…It’s just that technical part of it is kind of frustrating sometimes.”
		**No physical exam** (*n* = 13)	“Just let her check and see on her, especially since she has sickle cell, I want her to feel her spleen and stuff like that. You can’t do all of that stuff virtually.” “The only thing I would say that I just don’t like about telemedicine is that it’s just not in person. I would rather have doctors’ appointments in person or to prescribe new medication...seeing the person. You could always chart down a weight and things like that. But for me, it’s just something about physically an in-person [visit], it gives you a sense of okay, like things are going to be easier.”

### Caregiver effects

#### Burden/stress

Caring for a child with a chronic medical condition can lead to significant caregiver stress [27]. When discussing the experience of caregivers caring for their child with SCD, the majority (*n* = 12) reported feeling overwhelmed with the initial diagnosis and ongoing care. Caregivers reported the intermittent, unpredictable nature of the disease and its complications, as well as the feeling of helplessness during times of illness, can increase the stress they experience.

#### Guilt/responsibility

Caregivers also identified feelings of guilt and personal responsibility surrounding their child’s diagnosis of SCD. Five of the sixteen caregivers felt as though it was their fault that they were unaware they and their partner carried the sickle cell trait ultimately inherited by their child.

“I was really sad because I know that he’s going to have to deal with it for the rest of his life, and there’s nothing that I can really do, and then I felt kind of responsible for it because I didn’t know that I had the trait, and I didn’t know that his father had the trait, so I kind of felt responsible...like it was my fault that my son has this disease.”

#### Mental health

The increased burden and stress caregivers experience as a result of caring for a child with a chronic disease has been shown to be associated with increased mental health concerns for the caregiver [28–35]. Six of the sixteen caregivers in our study expressed concern about how their personal mental health has suffered.

### Barriers to care

#### Financial/distance

Caregivers were asked about both perceived and experienced barriers to accessing the necessary healthcare for their child. Common themes included the distance to the subspecialty SCD center and associated transportation needs, as well as financial strain and missed days of work. Fourteen of sixteen caregivers cited at least one of these areas as a barrier. Eleven caregivers also reported missing school as a hardship for their child that is associated with accessing needed SCD care.

#### Provider knowledge/comfort

Lack of provider knowledge and comfort associated with sickle cell care has been discussed previously, both amongst primary care providers and ED providers [36]. The majority (*n* = 9) of caregivers identified this as a concern within the general community, such as the school system, as well as within the medical system. Intertwined with this concern of provider knowledge and comfort regarding SCD, caregivers also reported the concern for provider bias [37].

### Facilitators to care

#### Convenience

Fourteen of the sixteen caregivers interviewed discussed how having subspecialists knowledgeable about SCD in the same space at the same time allowed for more convenient care, reducing the burden they experienced as a caregiver of a child with a chronic disease.

#### Dedicated sickle cell team/experts

Caregivers (*n* = 9) named access to hospital staff via phone/email and the ability to communicate with staff who are knowledgeable about SCD and familiar with their child as a facilitator of accessing SCD care. They appreciated being able to access a team directly that is dedicated to SCD, and felt this also led to prompt responses/solutions.

### View of telemedicine

#### Improves access

Telemedicine has been shown to improve access to care for children with complex medical needs who live in rural or medically under-resourced areas. Due to the ongoing COVID-19 pandemic, many patients and families, including our population, have participated in either hub-and-spoke or direct-to-consumer models of telemedicine. Hub-and-spoke telemedicine involves a specialist at a tertiary care center (hub) meeting virtually with the patient who is present at a local clinic site (spoke), whereas the direct-to-consumer model, which is the predominant form of telemedicine used during the current pandemic, connects the specialist virtually to the patient directly in their home. Most caregivers in our study stated telemedicine was beneficial, as it removes the barrier of distance/transportation to regular visits. Many also acknowledged the flexibility of telemedicine to allow for visits should there be difficulty for the family to be seen in person, or in the case of a pandemic, during spikes in cases.

#### Technology

Only 4 out of 16 caregivers who had participated in direct-to-consumer telemedicine mentioned difficulty with technology as a reason they would not want to regularly utilize this form of care delivery.

“A lot of times we do it on our phone and if you receive a call or something, you're kicked out. Then they have to get back on and redo everything…It's just that technical part of it is kind of frustrating sometimes.”

#### Lack of physical exam

Thirteen out of sixteen caregivers, all of whom had participated in direct-to-consumer telemedicine, cited the lack of a physical exam as a major reason why they did not prefer that model of telemedicine for visits. Caregivers reported they had a greater sense of reassurance that their child was well when an in-person exam was performed.

## Discussion

The lack of subspecialty centers for SCD compared to other genetic diseases has been well-documented [6, 38]. Fewer than 70% of children with SCD receive comprehensive subspecialty care, and only 20% experience effective care coordination between primary and subspecialty providers [39]. Although Crosby et al., have reported barriers to care experienced by individuals living with SCD, the effect of living in a largely rural midwestern state with medically under-resourced urban areas has not been well-described [15]. This qualitative study explored the experiences of caregivers in accessing healthcare for their child with SCD in rural and/or medically under-resourced areas of a midwestern state, as well as the use of telemedicine to overcome potential barriers.

The themes that emerged from the caregiver interviews in this study were consistent with barriers identified in previous research. However, these interviews revealed provider knowledge and bias as a novel, yet significant, barrier to receiving adequate SCD care. Caregivers often mentioned that they worry about how knowledgeable school personnel or a healthcare provider may be regarding SCD, and how this not only affects them personally, but also impacts their overall experience in the healthcare system. Caregivers expressed losing trust in their local community and hospitals because of this lack of knowledge, or worse, blatant bias (e.g., “…called drug seekers…”).

Trust of medical providers and the healthcare system is essential for ongoing care and shared decision-making in chronic diseases like SCD. It is especially important to build trust with patients with SCD and their caregivers, given that they generally comprise a particularly vulnerable population which has long been neglected and subject to racism in the healthcare system. Lack of provider knowledge or comfort with managing SCD not only increases this vulnerability and caregiver burden, but also furthers the development of medical mistrust. A focus on improving provider knowledge and addressing bias in the care of patients with SCD is essential to reducing barriers to accessing medical care [37]. Standardization of care through sickle cell-specific protocols, personalized pain plans, and broad provider education can address some of these concerns.

Another specific theme that caregivers frequently mentioned was related to long-term stress and mental health burden. Caregiver stress and the development or worsening of mental health has been well-documented, including in other chronic disease populations such as cystic fibrosis [35]. Additionally, caregiver perceptions of their child’s stress are associated with increased caregiver burden and worsening mental health [34]. Caregivers in our study reported worsening of their own mental health due to their child having a chronic disease, as well as the challenges associated with accessing care for their child. Thus, not only are they enduring the stress and burden of caring for a child with a lifelong illness, but also the structural inequities that lead to poorer access to care or quality of care adds to their burden and overall mental well-being.

To improve the access patients with SCD and their caregivers have to subspecialists with expertise in SCD, telemedicine has been piloted in similar rural and medically under-resourced communities [18]. The COVID-19 pandemic led to the mass adoption of the direct-to-consumer model of telemedicine, where the healthcare provider could deliver medical services directly to the patient in their home, often via a smartphone or tablet. This greatly increased the general population’s exposure to telemedicine, including individuals living with SCD. In our interviews, many of the caregivers had participated in some model of telemedicine. Interestingly, most who had experienced the direct-to-consumer model of telemedicine delivery thought a significant disadvantage was the lack of a hands-on physical exam. They viewed this disadvantage as significant enough that they would prefer to travel the distance to the subspecialty center to receive personalized care, despite the barriers that exist. The caregivers who had participated in the hub-and-spoke model of telemedicine for SCD care, did not report this as a disadvantage.

The barriers that caregivers of children with SCD experience in the Midwest have similarities to those previously reported in other parts of the U.S, and while telemedicine has been a useful adjunct to medical care during the COVID-19 pandemic, much still needs to be improved to provide adequate subspecialized care for the SCD population. Likely, there is value to leveraging multiple models of telemedicine (e.g., hub-and-spoke and direct-to-consumer) depending on the needs of the patient/caregiver, location, and disease severity. It may improve access to subspecialty care, but more importantly, similar to what has been done in pediatric diabetes care, these models need to be adapted to specifically serve the SCD population [40]. Further work evaluating adapted models for SCD care is needed to improve access to healthcare for this patient population.

Like all studies, this one has limitations that should be considered. The small, focused sample size representing a population in the Midwest can limit generalizability outside of the region. However, the sample size was adequate to achieve thematic saturation, demonstrating consistency in reported themes [41]. The study was also limited to caregivers of children and adolescents with SCD, and future studies evaluating perceptions and experiences of adolescent and young adult SCD patients living in rural or medically under-resourced areas would be of importance.

## Conclusion

This qualitative study evaluates the experiences and perceptions of barriers to accessing SCD care for caregivers living in rural and medically under-resourced areas. While some reported themes overlapped with previous qualitative studies, those related to caregiver burden and provider knowledge or biases suggest barriers to care that may be unique to SCD patients, requiring interventions and models of care to be adapted for this specific population.

## Data Availability

All data generated or analyzed during this study are included in this published article.

## Abbreviations

SCD: Sickle Cell Disease
ED: Emergency Department
BCQ: Barriers to Care Questionnaire
